# 

**Published:** 1887-08

**Authors:** 


WHOLE NUMBER CCCXXV. | AUGUST, 1887. | NUMBER 1. VOLUME ~~XXVIII~~ 27

### THE BUFFALO MEDICAL AND SURGICAL JOURNAL

ESTABLISHED YEAR 1844.

**EDITORS:**

**THOS. LOTHROP, M. D.** **A. R. DAVIDSON, M. D.**

**ASSOCIATE EDITORS:**

FLOYD S. CREGO, M. D. CARLTON C. FREDERICK, M. D.

FRED. R. CAMPBELL, M. D. FRANK H. POTTER, M. D.

EDWARD B. ANGELL, M. D., Rochester, N. Y.

